# Efficacy and safety of patients with chronic kidney disease undergoing left atrial appendage closure for atrial fibrillation

**DOI:** 10.1371/journal.pone.0287928

**Published:** 2023-10-26

**Authors:** Chaofan Liu, Shaojie Han, Kaijun Cui, Fang Wang

**Affiliations:** 1 School of Nursing, Chengdu University of Traditional Chinese Medicine, Chengdu, China; 2 Department of Cardiology, West China Hospital, Sichuan University, Chengdu, China; 3 Guang’an Shi Zhongyi Yiyuan: Guang’an Hospital of Traditional Chinese Medicine, Beijing, China; Hamad Medical Corporation, QATAR

## Abstract

**Background:**

The relative safety and efficacy of left atrial appendage closure (LAAC) for atrial fibrillation (AF) in patients with chronic kidney disease (CKD) have not been well defined. To evaluate the results in this cohort, we conducted a systematic review and meta-analysis of observational studies.

**Methods:**

We searched the PubMed, EMBASE, Web of Science, and Cochrane Library databases from inception to January 2023 for all relevant studies. Our inclusion criteria were met by twelve observational studies that included 61324 patients altogether.

**Results:**

Compared with no CKD group, in-hospital mortality (OR: 2.84, 95% CI: 2.12–3.81, p<0.01, I^2^ = 0%), acute kidney injury (AKI) (OR: 4.39,95% CI:4.00–4.83, P<0.01, I^2^ = 3%), major bleeding events (OR: 1.44, 95% CI: 1.29–1.60, p<0.01 I^2^ = 0%), and pericardial effusion/tamponade (OR 1.30; 95% CI 1.13–1.51, p < 0.01; I^2^ = 0%) were more common in the CKD group, especially in patients with end-stage renal disease (ESRD). No significant difference was observed in the occurrence of stroke (OR: 1.24, 95% CI: 0.86–1.78, P = 0.25, I^2^ = 0%), LAAC success rates (OR: 1.02, 95% CI: 0.33–3.16, p = 0.97, I^2^ = 58%) and vascular access complications (OR: 1.13, 95% CI: 0.91–1.39, p = 0.28, I^2^ = 0%) between the two groups. During the follow-up, there was no difference in the risk of stroke between the two groups.

**Conclusions:**

CKD patients who receive LAAC have a greater risk of in-hospital mortality, AKI, pericardial effusion/tamponade, and major bleeding events than those without CKD, especially in patients with ESRD. No significant difference in the risk of stroke was found in the long-term follow-up after LAAC between the two groups, demonstrating a similar efficacy of LAAC to prevent stroke in CKD patients.

## Introduction

Atrial fibrillation (AF) is a common cardiac arrhythmia in patients with end-stage renal disease (ESRD) or chronic kidney disease (CKD) [1]. CKD and ESRD are regarded as independent risk factors for the development of AF. Patients with CKD or ESRD have a prevalence of nonvalvular atrial fibrillation (NVAF) that ranges from 13% to 27% [2, 3]. Large prospective studies have revealed that people with CKD may be more susceptible to developing AF [4]. Stroke and systemic embolism are complications closely related to the prognosis of atrial fibrillation. For this reason, in patients with an elevated risk of thrombosis, as typically determined by the CHA2DS2-Vasc score, current guidelines recommend oral anticoagulants for the prevention of embolic events [1]. Patients with impaired renal function not only had a higher incidence of AF but also had a higher risk of embolism than patients with normal renal function [5, 6]. In addition to the increased thromboembolic risk, patients with AF and concurrent CKD are more likely to experience bleeding events, especially if they are using anticoagulants [5, 7]. Moreover, due to a lack of data, novel oral anticoagulant (NOAC) administration should be avoided in patients with significantly impaired renal function, and the use of warfarin is linked to contradictory outcomes [1]. Over the past ten years, a non-pharmacologic method of preventing ischemic stroke in patients with AF who are not anticoagulant candidates has gained widespread acceptance as an alternative to NOACs. This method involves percutaneous left atrial appendage closure (LAAC) using the Watchman, Amplatzer Cardiac Plug, Lambre device and so on [8].

Several studies have evaluated the effectiveness and safety of LAAC in CKD patients, but these are mostly small-sample studies, and the results of different studies are conflicting. To better understand the safety and long-term efficacy of LAAC for patients with AF and CKD, we conducted a meta-analysis of the literature.

## Methods

The review was carried out in accordance with the Preferred Reporting Items for Systematic Reviews and Meta-Analyses standards [9].

### Search strategy

To discover all published clinical studies that reported the effects of LAAC in CKD or ESRD and no CKD or ESRD. A thorough review of the literature was performed in the PubMed, Web of Science, Cochrane Library, and Embase databases up to January 2023. Each term included “atrial fibrillation” and “left atrial appendage”, and (“renal Insufficiency” or “renal failure” or "end-stage renal disease" or "chronic kidney disease" or "dialysis" or "hemodialysis"). We have no restrictions on the type of research. A manual search of secondary materials, including the references of initially found articles, reviews, and comments, was used to find relevant studies. All references were downloaded in order to consolidate, get rid of duplicates, and conduct additional research.

### Study selection and outcomes

The search results were examined by two blinded, independent writers (C.L. and S.H.), who included papers if they followed the following standards: (1) peer-reviewed journals published the research, (2) compared patients receiving LAAC for AF who had CKD or ESRD to those who had not, and (3) at least one LAAC-related complication should be mentioned. No limitations on sample size, follow-up time, or language existed for us. We talked until we came to an agreement when there were differences between the reviewers. Cohort study quality was evaluated using the Newcastle-Ottawa quality assessment scale (NOS).

Interested procedural endpoints included in-hospital mortality, stroke, LAAC success rate, major bleeding events, vascular access complications, acute kidney injury, and pericardial effusion/tamponade. Long‐term endpoints included all‐cause mortality, stroke, and major bleeding events. Four of the included studies reported composite endpoint, but the definition of these composite endpoints was not exactly the same. We did not include them in the final analysis. Procedural success was defined as successful implantation and a peri-device leakage less than 5 mm based on our included studies. This is the definition given by the current guidelines and the studies we included, and this definition is the result of numerous previous studies [10].

Based on the studies we included, patients with estimated glomerular filtration rate (eGFR)<60 mL/min/1.73 m2 were categorized as having a chronic kidney disease (CKD), and end-stage renal disease (ESRD) was defined as the eGFR < 15 ml/min/1.73 m2 or chronic hemodialysis treatment [11, 12].

### Data extraction

C.L. and S.H. separately searched the articles and retrieved the data. When the two authors were unable to agree, the third author (F.W.) was approached to make a decision. Each study’s data were thoroughly extracted to yield the following information: title, name of the first author, publication year, year of the study, country, demographic and characteristic data of subjects, total numbers of participants, and complication rates in each study.

### Statistical analysis

Means and standard deviations for continuous variables and the quantity and percentages for categorical variables are how descriptive statistics are presented. The Cochrane Collaboration’s and PRISMA guidelines’ recommendations for statistical analysis were followed, using Review Manager, version 5.4. The effects on procedural and long‐term outcomes were presented as odds ratios (ORs) with 95% confidence intervals (CI). Regardless of heterogeneity among the studies, inverse variance weighted random-effects approach was adopted for each outcome since it allowed for a more conservative evaluation of the pooled effect size. A sensitivity analysis was conducted by removing one study at a time to analyze the impact of each study on overall heterogeneity and to assess the study’s overall robustness. According to Cochrane, publication bias was evaluated by looking at the funnel plot’s symmetry. The cutoff for statistical significance was p <0.05.

## Results

### Study characteristics

**Fig 1:**
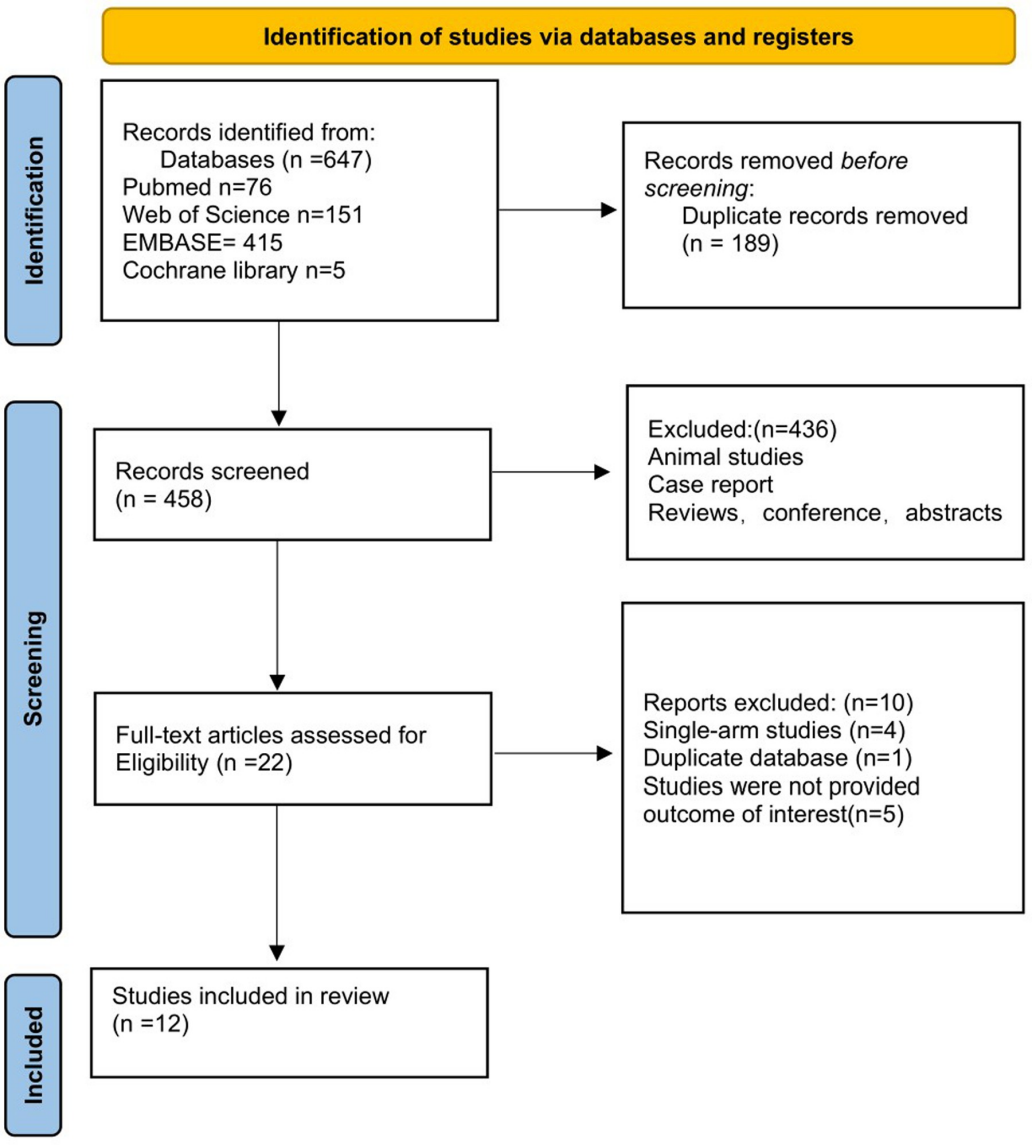
Flow chart of literature screening.

The initial results from our search method returned 647 potentially useful articles (415 articles from Embase, 151 articles from Web of Science,76 articles from PubMed, and 5 from Cochrane Library (Fig 1). 458 papers were subjected to title and abstract screening after 189 duplicate articles were eliminated. Twenty-two articles received full text analysis, and 436 papers were excluded. 4 studies were single-arm studies, 1 study was disregarded since it used the same database. 5 of the 22 publications were disregarded because their results were uninteresting. Finally, our research included 12 observational studies enrolling a total of 61324 patients who underwent LAAC [10–21]. Four studies utilized the Watchman device, one utilized the Amplatzer Cardiac Plug device, and 7 applied more than 3 types of devices, including the Watchman, Amplatzer cardiac plug, Amplatzer Amulet, Lambre, or Wavecrest device. The research’s characteristics are summarized in Table 1. The NOS scores of the studies varied from 6 to 8 and are shown in S1 Table in S1 File. Compared with the no CKD group, the CKD group was more likely to present with coronary heart disease (60.23% vs. 46.20%, p < 0.01), hypertension (61.37% vs. 58.27%, p < 0.01), diabetes (32.01% vs. 24.02%, p < 0.01), and stroke/ transient ischemic attack (TIA) (34.36% vs. 26.94%, p < 0.01). In addition, in the CKD group, there were higher CHA2DS2‐VASc scores and HAS‐BLED scores. The patient’s baseline characteristics are summarized in Table 2.

**Table 1:** Characteristics of the included studies.

Study	Country	Study design	Patient selection	Follow-up	Type of Occluder
Kefer et al. (2016)	Europe and North America	Prospective multicenter study	2008,12–2013,11	498d	1
Xue et al. (2018)	Germany	Single-centre, retrospective study	2012,2–2017,1	637d	2
Luani et al. (2019)	Germany	Single-centre, prospective study	NR	1.6y	3
Brockmeyer et al. (2020)	Germany	Single-centre, retrospective study	2012,3–2016,3	391.2d	4
Ahuja et al. (2021)	America	Retrospective multicenter study	2016,1–2017,12	90d	NR
Faroux et al. (2021)	Europe and North America	Retrospective multicenter study	NR	2y	5
Fastner et al. (2021)	Germany	Prospective multicenter study	2014,7–2016,1	More than 1y	NR
Munir et al. (2021)	America	Retrospective multicenter study	2015–2018	NR	6
Benini et al. (2022)	Spain	Single-centre, retrospective study	2011,1–2019,7	567d	7
Michlicka-Klys et al. (2022)	Poland	Single-centre, prospective study	2009–2019	25.56m	8
Ueno et al. (2022)	Japan	Single-centre, retrospective study	2020,6–2022,4	45d	9
Fink et al. (2023)	Germany	Retrospective multicenter study	2006,4–2019,12	405d	10

1 Amplatzer Cardiac Plug; 2 Watchman; 3 Watchman; 4. Amplatzer Cardiac Plug, Amulet, and Watchman, 5. Amplatzer Cardiac Plug, Amulet, Watchman, Lambre, and Ultrasea device, 6 Watchman 7 ACP, Amulet, Watchman, and Others;8 ACP, Amulet, and Watchman; 9 Watchman; 10 Watchman, Watchman FLX, AMPLATZER Amulet, Lambre, Wavecrest Device; NR:not report.

**Table 2:** Characteristics of the included patients.

Study	Group	Sample, N	Male, N (%)	Mean, Age	CHA2DS2‐VASc score	HAS‐BLED score	HTN, N (%)	DM, N (%)	Stroke/TIA, N (%)	CAD, N (%)
Kefer et al. (2016)	Non-CKD	639	425(66.5)	74.9±8.4	4.2±1.6	2.9±1.1	536(83.9)	152(23.8)	264(41.3)	183(28.6)
	CKD	356	194(54.5)	78.0±7.4	4.9±1.5	3.4±1.2	321(90.2)	135(37.9)	111(31.2)	162(45.5)
	ESRD	19	11(57.9)	76.9±6.6	4.7±1.7	4.3±1.3	13(68.4)	8(42.1)	9(47.4)	9(47.4)
Xue et al. (2018)	Non-CKD	149	111(74.5)	73.2±7.8	3.4±1.4	3.0±1.0	115(77.2)	29(19.5)	22(14.8)	NR
	CKD	151	92(60.9)	77.0±7.2	4.3±1.5	4.0±1.0	123(81.5)	56(37.1)	17(11.3)	NR
Luani et al. (2019)	Non-CKD	116	74(63.8)	72.1±8.0	3.4±1.6	3.1±0.9	98(84.5)	40(34.5)	21(17.5)	NR
	CKD	73	42(57.5)	75.9±6.7	4.5±1.4	3.7±1.0	66(90.4)	45(61.6)	12(16.4)	NR
Brockmeyer, et al. (2020)	Non-CKD	65	42(64.6)	74.4±7.1	4.1±1.4	3.7±0.8	55(84.6)	19(29.2)	14(21.5)	42(64.6)
	CKD	81	42(51.9)	78.2±7.3	4.7±1.3	3.9±0.9	76(93.8)	33(40.7)	14(17.3)	63(77.8)
Ahuja et al. (2021)	Non-CKD	16749	10214(61.0)	73.6±9.4	3.6±1.5	NR	10391(62.0)	4733(28.3)	NR	7816(56.7)
	CKD	3954	2555(64.6)	76.1±8.1	4.2±1.4	NR	2027(51.3)	1855(46.9)	NR	2426(61.4)
	ESRD	571	375(65.7)	69.5±20.9	3.8±1.4	NR	351(61.6)	333(58.3)	NR	358(62.8)
Faroux et al. (2021)	Non-CKD	794	486(61.2)	75.1±8.4	4.4±1.5	3.5±1.0	669(84.3)	245(30.9)	303(38.2)	238(30.0)
	CKD	300	184(61.3)	77.8±8.2	4.9±1.5	4.0±1.1	283(94.3)	134(44.7)	95(31.7)	124(41.3)
Fastner et al. (2021)	Non-CKD	324	218(67.3)	76(71–80)	4.2±1.5	3.5±1.0	301(92.9)	84(25.9)	105(32.4)	123(38.0)
	CKD	284	150(52.8)	NR	4.9±1.4	4.3±1.0	265(93.3)	120(42.3)	75(26.4)	155(54.6)
	ESRD	15	12(80.0)	75(69–79)	5.1±1.7	4.6±1.1	14(93.3)	10(66.7)	4(26.7)	11(73.3)
Munir et al. (2021)	Non-CKD	31405	18005(57.3)	77(71–82)	4(3–4)	NR	17060(54.3)	6735(21.4)	NR	14690(46.8)
	CKD	3545	2285(64.5)	77.5(73–83)	4(3–4)	NR	2127(60.0)	185(5.2)	NR	2130(60.1)
	ESRD	1115	755(67.7)	71(65–78)	3(2–4)	NR	724(65.0)	40(3.6)	NR	730(65.5)
Benini et al. (2022)	Non-CKD	53	32(60.4)	73(65–78)	4(3–5)	3(3–4)	47(88.3)	11(21.8)	25(59.9)	NR
	CKD	71	45(63.4)	77(69–81)	4(3–6)	4(3–4)	65(91.6)	23(32.4)	24(43.6)	NR
Michlicka-Klys, et al. (2022)	Non-CKD	167	102(61.1)	71.0±9.1	3.7±1.4	2.9±0.7	NR	47(28.1)	37(22.2)	NR
	CKD	105	49(46.7)	75.5±7.7	4.9±1.3	3.2±0.9	NR	41(39.0)	27(25.7)	NR
Ueno et al. (2022)	Non-ESRD	93	61(65.6)	80(75–84)	5(4–6)	3(2–3)	71(76.3)	26(28.0)	39(41.9)	NR
	ESRD	25	20(80.0)	73(71–79)	5(4–6)	4(3–5)	16(64.0)	12(48.0)	16(64.0)	NR
Fink et al. (2023)	Non-ESRD	57	34(59.6)	73.5±8.6	4.8±1.6	3.0±1.1	53(93.0)	35(61.4)	14(24.6)	NR
	ESRD	57	40(70.2)	73.9±7.4	4.6±1.7	3.5±1.0	54(94.7)	30(52.6)	10(17.5)	NR

HTN: hypertension; DM: diabetes; TIA: transient ischemic attack; CAD: coronary artery disease; CKD: chronic kidney disease; ESRD: end-stage renal diseas

### Procedural outcomes

**Fig 2:**
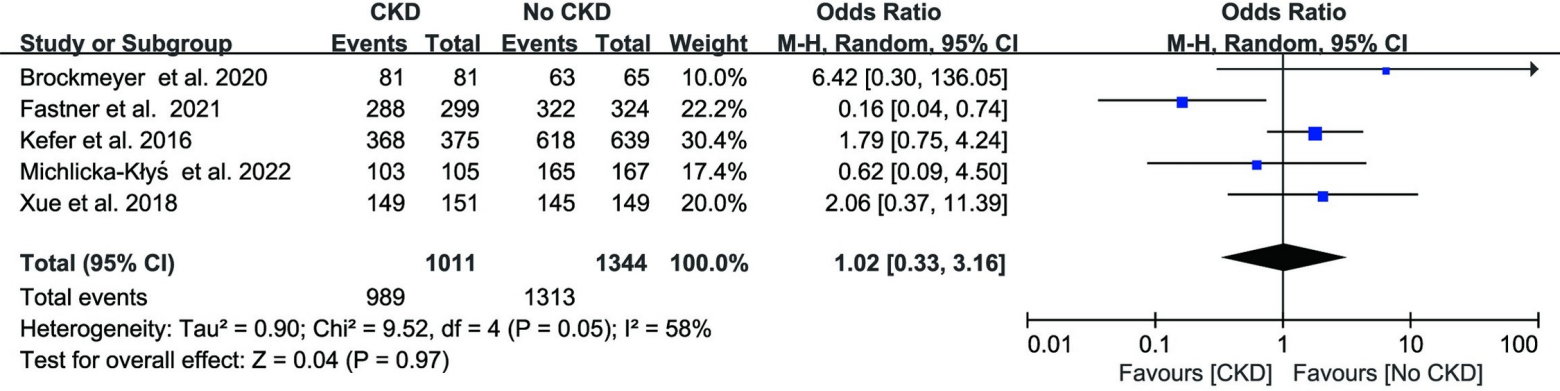
Forest plots showing the effect of LAAC success rate in patients with or without chronic kidney disease.

The LAAC success rates were reported in five studies, and they all failed to discover a connection between CKD and LAAC success rates (OR: 1.02, 95% CI: 0.33–3.16, p = 0.97 Fig 2). Modern heterogeneity was present across the analyses (I^2^ = 58%, p = 0.05). The outcomes are displayed in Fig 2. In addition, we separately analyzed in-hospital mortality, stroke, pericardial effect/tamponade, vascular access complications, and major bleeding events associated with the procedure.

**Fig 3:**
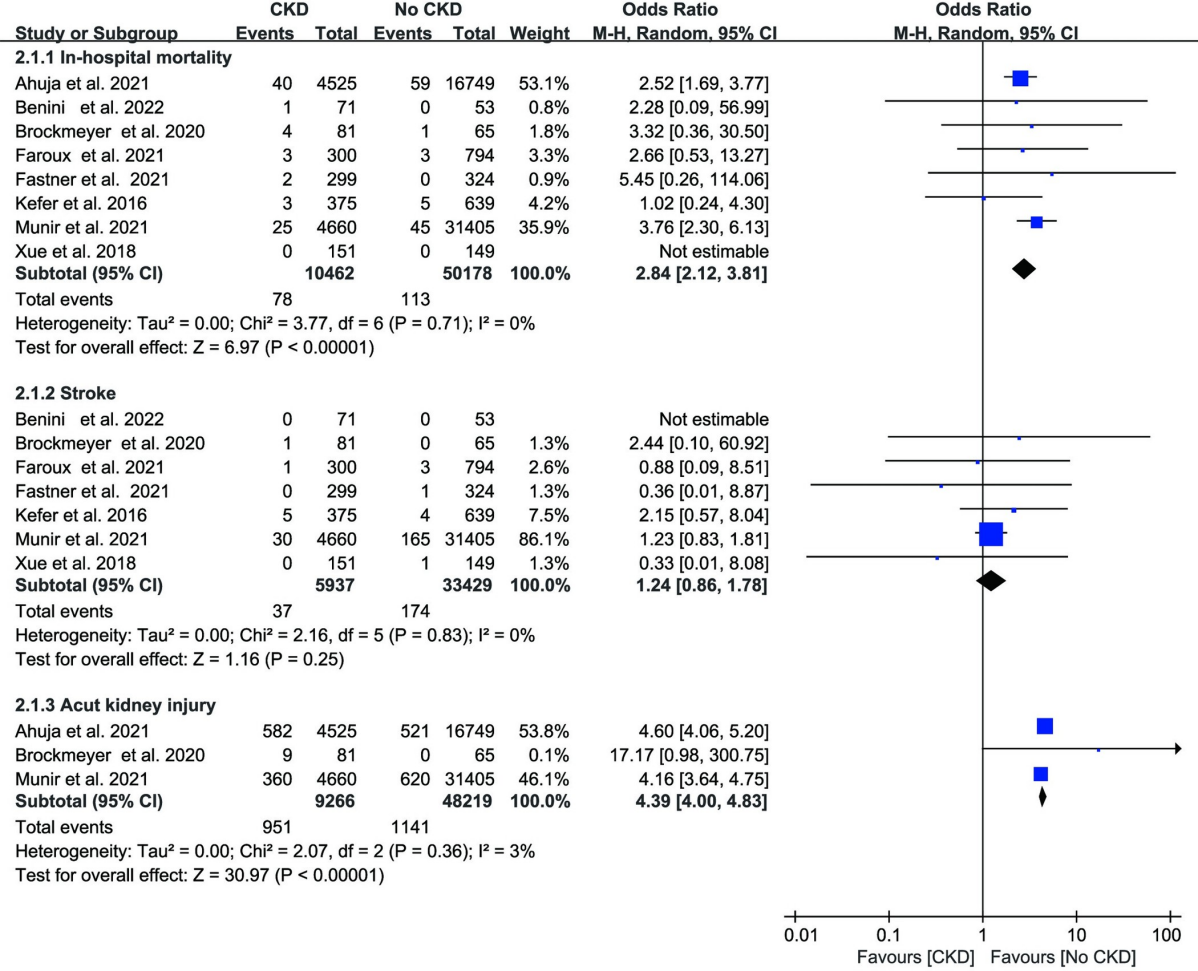
Forest plots showing the effect of in-hospital mortality, stroke and acute kidney injury in patients with or without chronic kidney disease.

As shown in Fig 3, data on in-hospital mortality were provided by eight studies, totaling 60650 patients. CKD increased the risk of in-hospital mortality by 2.84 times according to the overall meta-analysis (OR: 2.84, 95% CI: 2.12–3.81, p < 0.01). Low heterogeneity was present across the studies (I^2^ = 0%, p = 0.71). In addition, we discovered that individuals with ESRD had an 8.61-fold higher rate of in-hospital mortality than those without ESRD (OR: 8.61, 95% CI: 5.94–12.48, p < 0.01 I^2^ = 0%, Fig 5).

**Fig 4:**
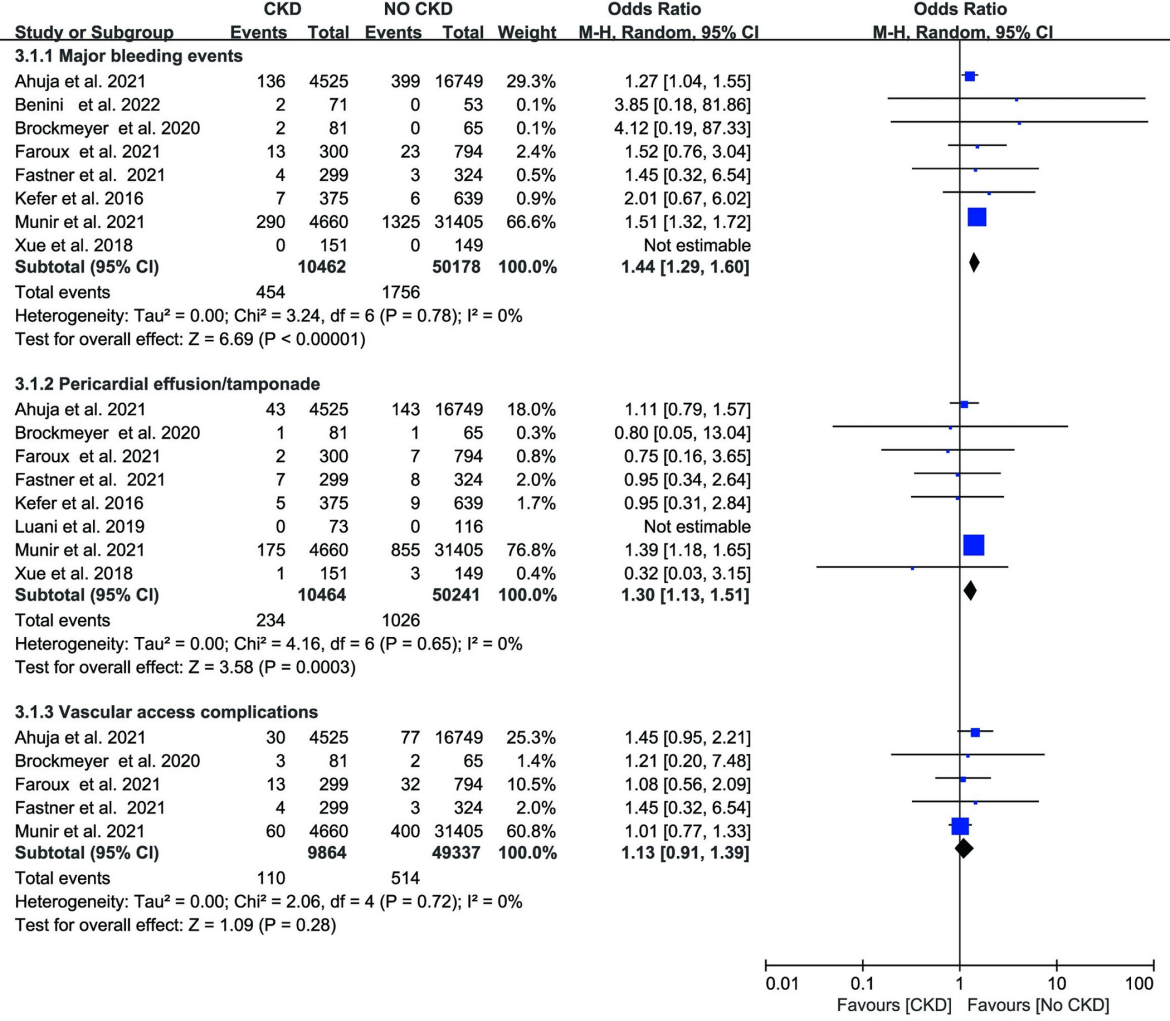
Forest plots showing the effect of major bleeding events, pericardial effusion/tamponade and vascular access complications in patients with or without chronic kidney disease.

**Fig 5:**
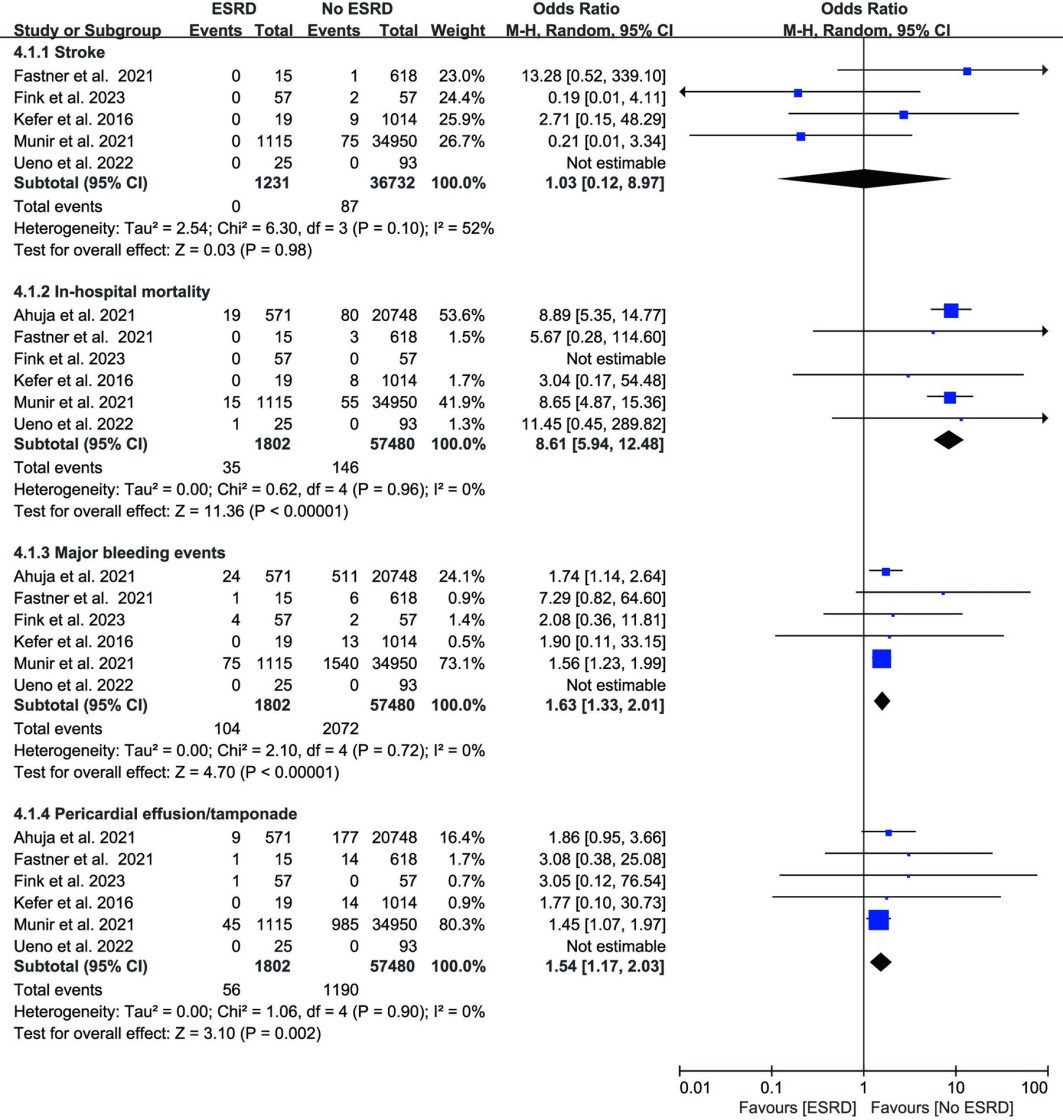
Forest plots showing the effect of stroke, in-hospital mortality, major bleeding events and pericardial effusion/tamponade in patients with or without end-stage renal disease.

Seven studies reported stroke cases, and the two groups did not differ significantly (OR: 1.24, 95% CI: 0.86–1.78, p = 0.25, I^2^ = 0%, Fig 3). In three investigations, acute kidney injury (AKI) was mentioned. The CKD group had a greater incidence (OR: 4.39, 95% CI: 4.00–4.83, p <0.01, I^2^ = 3%, Fig 3). Periprocedural major bleeding events were documented in eight investigations. The meta-analysis showed a 1.44-fold increased incidence of major bleeding events in people with CKD (OR: 1.44, 95% CI: 1.29–1.60, p < 0.01, Fig 4). No evidence of heterogeneity was found among the studies (I^2^ = 0%, p = 0.32). In the ESRD group, patients had a 1.6-fold risk of bleeding (OR: 1.63, 95% CI: 1.33–2.01, p < 0.01, I^2^ = 3%, Fig 5). Vascular access complications were reported in 5 studies. Between the CKD group and the no CKD group, there was no statistically significant difference in the frequency of vascular access complications (OR: 1.13, 95% CI: 0.91–1.39, p = 0.28 I^2^ = 0%, Fig 4). Additionally, our research revealed that pericardial effusion/tamponade in the CKD group was significantly higher than in the no CKD group (OR 1.30; 95% CI 1.13–1.51, p < 0.01; I^2^ = 0%, Fig 4). Likewise, patients with ESRD showed a greater incidence of pericardial effusion/tamponade (OR: 1.54, 95% CI: 1.17–2.03, p < 0.01, I^2^ = 0% Fig 5).

### Long‐term outcomes

**Fig 6:**
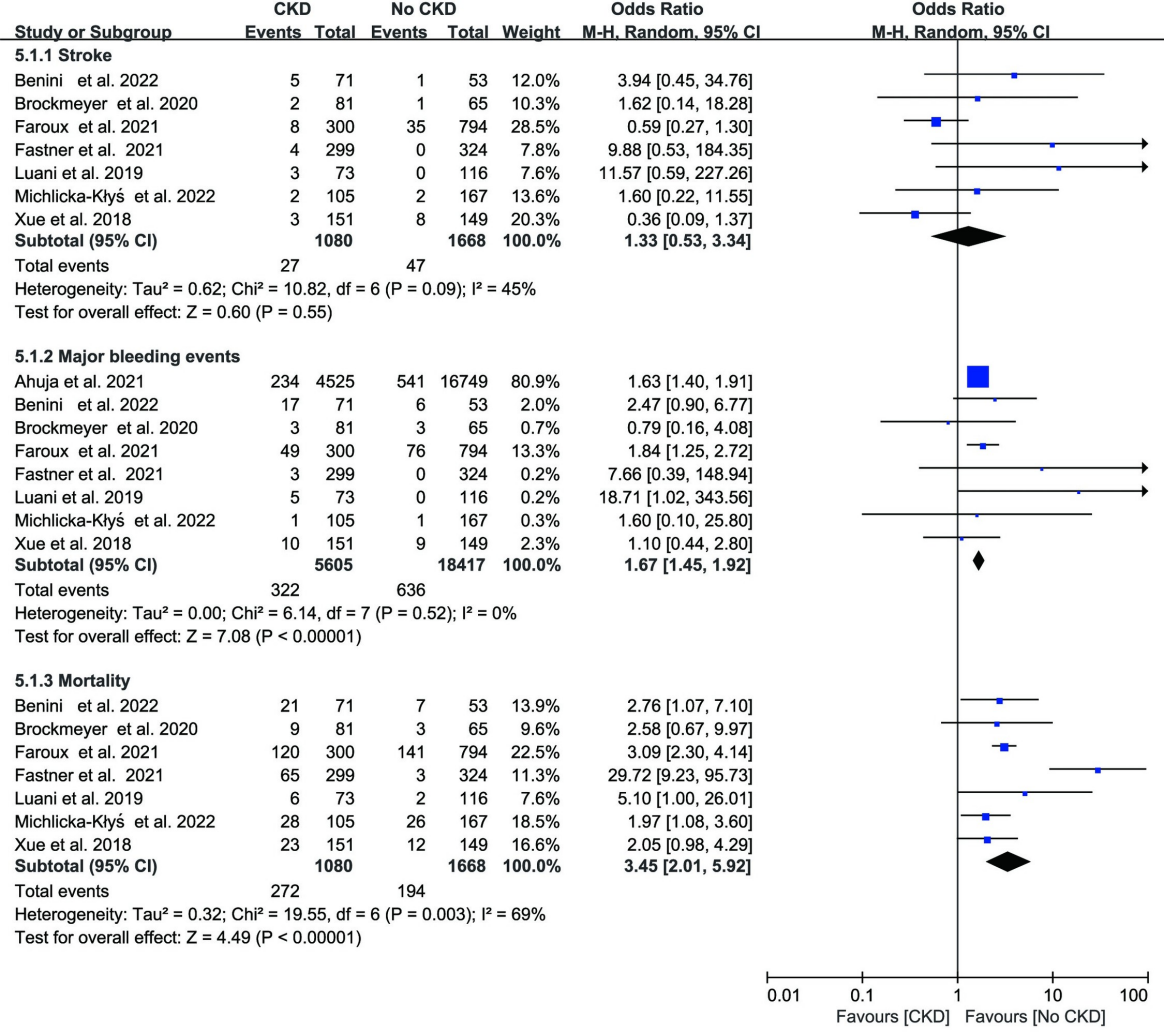
Forest plot showing the effect of left atrial appendage closure on the overall risk of stroke, bleeding, and mortality in the long-term follow up.

Seven studies, with an average follow-up period of 1–2 years, described the outcomes of long-term follow-up following LAAC. During the follow-up, there was no discernible difference in the risk of stroke between the CKD group and the no CKD group (OR 1.33; 95% CI 0.53–3.34; p = 0.55; I^2^ = 45%; Fig 6). The CKD group had higher major bleeding events during follow-up than the no CKD group (OR: 1.67; 95% CI: 1.45–1.92; p < 0.01; I^2^ = 0%; Fig 6). During the follow-up, the CKD group’s all-cause mortality was greater than that of the group without CKD (OR 3.45; 95% CI 2.01–5.92; p < 0.01; I^2^ = 69%, Fig 6).

### Sensitivity analysis

To determine how each study’s removal influenced the results, we carried out a sensitivity analysis. An overview of the outcomes of the sensitivity analysis is given in S2 Table in S1 File. The result of pericardial effusion/tamponade between the CKD and no CKD groups exhibited poor stability in the sensitivity analysis, which was done by deleting the greatest sample size. After excluding the study by Munir et al., the pericardial effusion/tamponade rates were comparable between CKD and no CKD (OR: 1.44, 95% CI: 0.69–3.00, p = 0.34). The remaining endpoints showed good stability in the sensitivity analysis. Given the number of studies, we included in our analysis, we did not identify publication bias.

## Discussion

The following are the key conclusions of our study: 1) the current study shows that patients with and without CKD experience similar procedure success rates. In the perioperative period, the CKD and non-CKD groups had similar risks of stroke and vascular access complications. 2) patients with CKD tended to have more periprocedural pericardial effect/tamponade, major bleedings, in-hospital mortality, and AKI after LAAC. 3) the data also revealed that after a long follow-up, the stroke rates for patients with and without CKD were the same.

It is still challenging to treat patients with AF who have CKD or ESRD. Compared to those without CKD, patients with AF and CKD had a higher risk of stroke and bleeding incidents [5]. Patients with ESRD and AF provide additional clinical challenges because there is little evidence to show a reduction in thromboembolic events associated with OACs while bleeding incidents are still common [22, 23].

Our research revealed that the CKD group had an almost 3-fold higher in-hospital mortality rate, while the risk of in-hospital mortality increased 8-fold in patients with ESRD. In the general population, CKD is independently linked to death [24]. AF and CKD are closely related diseases with a shared set of risk factors such as coronary artery disease and hypertension [25, 26]. Additionally, CKD and ESRD have been linked to higher mortality and morbidity rates in patients undergoing percutaneous coronary intervention and transcatheter aortic valve replacement [27, 28]. Prior small-scale studies found no differences between patients with and without CKD in in-hospital mortality. These studies, however, had very few or no patients with ESRD and their sample sizes were constrained [13, 15, 18, 20]. According to our findings, in-hospital mortality was significantly higher in CKD/ESRD patients than in patients without CKD/ESRD patients. Based on other studies of structural heart interventions, these people have a higher chance of dying from cardiac interventions because they are sicker at the start of treatment [27, 28]. Regrettably, no specific cause of death was given in our included studies.

After LAAC, patients with CKD experienced AKI more frequently than patients without CKD. To see the LAA before and after closure during LAAC, contrast is routinely used. Approximately 10% to 13% of patients undergoing LAAC have reported AKI [29–31]. AKI after LAAC has previously been reported to be independently correlated with CKD [31]. Furthermore, AKI after LAAC may cause thromboembolic effects, such as stroke. To prevent AKI, efforts should be made to maximize protect renal function, reduce contrast volume, and prevent sudden hemodynamic changes like hypotension during surgery. Patients with CKD experienced a greater incidence of bleeding events in our study during the perioperative period. Patients with AF and concurrent CKD are more likely to experience bleeding events. A significant retrospective cohort study with 516197 patients indicated that for every 10 mL/min/1.73 m^2^ decrease in eGFR, the chance of hemorrhaging increased by 9% [32]. Compared to patients without CKD, those with CKD had a slightly higher HAS-BLED score. The higher overall bleeding risk may help to explain some of the higher bleeding rates. Therefore, our study underscores the significance of the link between renal disorders and stroke risk in patients with AF and extends these findings to patients with CKD receiving LAAC. In addition, we found a higher incidence of pericardial effusion/ tamponade in CKD patients, but after removing the largest study by sensitivity analysis, the results were found to change, and the incidence between the two groups was not significantly different. Further studies may be needed in the future. However, in patients with ESRD, we found a higher incidence of pericardial effusion/ tamponade than in no ESRD patients.

CKD patients are more likely to experience thromboembolic events [33]. Despite patients with CKD having a considerably increased risk of stroke as indicated by their CHA2DS2-VASc score compared to those without CKD, we observed comparable rates of stroke in both groups following the effective LAAC. Furthermore, we discovered a 96.4% decrease in the risk of ischemic stroke in the CKD group and a 91.8% decrease in the no CKD group when compared to the projected risk based on the data released by Lip et al.[34] These results all indicate that LAAC is effective in preventing stroke in patients with CKD. During long-term follow-up, CKD patients had more bleeding events. More than half of the research we included was gastrointestinal hemorrhage. In fact, regardless of other variables like antithrombotic medication, the risk of gastrointestinal bleeding rises as eGFR declines [35]. In individuals with unexplained gastrointestinal bleeding, CKD is also linked to a higher risk of recurrent bleeding [36]. The greater rate of bleeding seen in these patients, particularly at the start of follow-up, may be related to the fact that LAAC requires anticoagulant medication for at least three months following the LAAC [37]. According to several studies, three months after LAAC, approximately 50% of bleeding incidents occur [20, 38]. During the follow-up period, the CKD group had a higher incidence of all-cause mortality. This could be explained in part by the fact that CKD patients were older and had more comorbid conditions than non-CKD patients, particularly cardiovascular disorders. Furthermore, a study has shown, that patients with CKD already have a lower life expectancy than the general population [24].

The studies we included spanned from 2016 to 2023, and there is no uniform regulation on anticoagulation after LAAC. Among the studies we included, 6 studies gave detailed anticoagulation recommendations after LAAC [10–12, 14, 20, 21]. But only one study gave the number of people using each anticoagulant or antiplatelet drug. In Fink et al. ’s study, there was a higher rate of use of dual antiplatelet (84.2% vs. 64.9%) and a lower rate use of anticoagulant (12.3% vs.35.1%) in the end-stage renal disease (ESRD) group compared with the non-ESRD group [12].

### Limitations

First, because the included studies are observational, confounders and bias risk do exist. Despite having the most reliable data, any conclusions reached depend greatly on the bias risk of the individual research and should be treated with caution. Second, we lacked access to individual patient data, and our use of the available summary data from published research was constrained. Third, we found a higher incidence of pericardial effusion in CKD patients, but after removing the largest study by sensitivity analysis, the results were found to change, and the incidence between the two groups was not significantly different. Further studies may be needed in the future.

## Conclusions

In conclusion, although the success rates of LAAC are comparable between the two groups, CKD patients who receive LAAC have a greater risk of in-hospital mortality, AKI, and bleeding events than those without CKD, especially in patients with ESRD. No significant difference in the risk of stroke was found in the long-term follow-up after LAAC between the two groups, demonstrating a similar efficacy of LAAC to prevent stroke in CKD patients.

## Supporting information

S1 Checklist (DOCX)

S1 File Search strategies. (DOCX)
