# Supplementary material for: Efficacy and safety of patients with chronic kidney disease undergoing left atrial appendage closure for atrial fibrillation
Source: PLoS One. 2023 Oct 26;18(10):e0287928. doi: 10.1371/journal.pone.0287928 (PMC10602315; doi:10.1371/journal.pone.0287928)
Supplement: S1 File — (DOCX) [file pone.0287928.s002.docx]

Search Strategies

#1 "Atrial Fibrillation"[Mesh] OR "atrial fibrillation"[Title/Abstract]

#2 "Atrial Appendage"[Mesh] OR "Left Atrial Appendage"[Title/Abstract]

#3"Renal Dialysis"[MeSH] OR "Renal Insufficiency"[MeSH] OR "end stage renal disease"[Title/Abstract] OR "chronic kidney disease"[Title/Abstract] OR "dialysis"[ Title/Abstract] OR Hemodialysis [Title/Abstract]

#4 #1 AND #2 AND #3

Supplement Table 1 Risk of bias assessment

| Study ID | Selection | Comparability | Outcomes | NOS score |
| --- | --- | --- | --- | --- |
| Kefer, J., et al. (2016) | ⭐⭐⭐⭐ | ⭐ | ⭐⭐⭐ | 8 |
| Xue, X., et al. (2018) | ⭐⭐⭐ | ⭐ | ⭐⭐ | 6 |
| Luani, B., et al. (2019) | ⭐⭐⭐⭐ | ⭐ | ⭐⭐⭐ | 8 |
| Brockmeyer, M., et al. (2020) | ⭐⭐⭐ | ⭐ | ⭐⭐ | 6 |
| Ahuja, K. R., et al. (2021) | ⭐⭐⭐ | ⭐ | ⭐⭐ | 6 |
| Faroux, L., et al. (2021) | ⭐⭐⭐ | ⭐ | ⭐⭐ | 6 |
| Fastner, C., et al. (2021) | ⭐⭐⭐⭐ | ⭐ | ⭐⭐⭐ | 8 |
| Munir, M. B., et al. (2021) | ⭐⭐⭐ | ⭐ | ⭐⭐ | 6 |
| Benini Tapias, J., et al. (2022) | ⭐⭐⭐ | ⭐ | ⭐⭐ | 6 |
| Michlicka-Klys,W.,et al. (2022) | ⭐⭐⭐⭐ | ⭐ | ⭐⭐⭐ | 8 |
| Ueno, H., et al. (2022) | ⭐⭐⭐ | ⭐ | ⭐⭐ | 6 |
| Fink, T., et al. (2023) | ⭐⭐⭐ | ⭐⭐ | ⭐⭐⭐ | 8 |

（⭐）Asterisks denote the quality of each domain. Numbers of stars in good quality:3 or 4 in selection, 1 or 2 in comparability and 2 or 3 in outcomes. Numbers of stars in fair quality:2 in selection,1 or 2 in comparability and 2 or 3 in outcomes. Numbers of stars in poor quality:0 or 1 in selection,0 in comparability, and 0 or 1 in outcomes.

NOS, Newcastle-Ottawa scale.

Supplement Table 2: Sensitivity Analysis

| **Study Excluded** | **Odds Ratio (95% Confidence Interval)** | ***p-*value** | ***I^2^*(%)** |
| --- | --- | --- | --- |
| **Left atrial appendage closure success rate** | | | |
| \| Brockmeyer et al. 2020 \| \| --- \| | 0.83（0.25-2.78） | 0.76 | 64 |
| \| Fastner et al. 2021 \| \| --- \| | 1.71（0.85-3.45） | 0.13 | 0 |
| \| Kefer et al. 2016 \| \| --- \| | 0.84（0.18-3.78） | 0.82 | 58 |
| \| Michlicka-Kłyś et al.2022 \| \| --- \| | 1.15（0.29-4.62） | 0.84 | 67 |
| \| Xue et al. 2018 \| \| --- \| | 0.87（0.21-3.62） | 0.84 | 66 |
| **In-hospital mortality** | | | |
| \| Ahuja et al. 2021 \| \| --- \| | 3.25（2.12-4.98） | <0.001 | 0 |
| \| Benini et al. 2022 \| \| --- \| | 2.85（2.12-3.82） | <0.001 | 0 |
| \| Brockmeyer et al. 2020 \| \| --- \| | 2.83（2.11-3.81） | <0.001 | 0 |
| \| Faroux et al. 2021 \| \| --- \| | 2.85（2.11-3.84） | <0.001 | 0 |
| \| Fastner et al. 2021 \| \| --- \| | 2.82（2.10-3.79） | <0.001 | 0 |
| \| Kefer et al. 2016 \| \| --- \| | 2.97（2.20-4.01） | <0.001 | 0 |
| \| Munir et al. 2021 \| \| --- \| | 2.43（1.68-3.50） | <0.001 | 0 |
| **Stroke** | | | |
| \| Brockmeyer et al. 2020 \| \| --- \| | 1.23(0.85-1.77) | 0.27 | 0 |
| \| Faroux et al. 2021 \| \| --- \| | 1.25(0.87-1.80) | 0.23 | 0 |
| \| Fastner et al. 2021 \| \| --- \| | 1.26(0.87-1.81) | 0.22 | 0 |
| \| Kefer et al. 2016 \| \| --- \| | 1.18(0.81-1.73) | 0.38 | 0 |
| \| Munir et al. 2021 \| \| --- \| | 1.32(0.50-3.48) | 0.58 | 0 |
| \| Xue et al. 2018 \| \| --- \| | 1.26(0.88-1.81) | 0.21 | 0 |
| **Acute kidney injury** | | | |
| Ahuja et al. 2021 | 4.17(3.65-4.77) | <0.001 | 0 |
| \| Brockmeyer et al. 2020 \| \| --- \| | 4.39(3.97-4.84) | <0.001 | 16 |
| \| Munir et al. 2021 \| \| --- \| | 4.61(4.07-5.21) | <0.001 | 0 |
| **Major bleeding events** | | | |
| \| Ahuja et al. 2021 \| \| --- \| | 1.52(1.34-1.72) | <0.001 | 0 |
| \| Benini et al. 2022 \| \| --- \| | 1.44(1.29-1.60) | <0.001 | 0 |
| \| Brockmeyer et al. 2020 \| \| --- \| | 1.44(1.29-1.60) | <0.001 | 0 |
| \| Faroux et al. 2021 \| \| --- \| | 1.44(1.29-1.60) | <0.001 | 0 |
| \| Fastner et al. 2021 \| \| --- \| | 1.44(1.29-1.60) | <0.001 | 0 |
| \| Kefer et al. 2016 \| \| --- \| | 1.44(1.29-1.60) | <0.001 | 0 |
| \| Munir et al. 2021 \| \| --- \| | 1.32(1.09-1.58) | 0.004 | 0 |
| **Pericardial effusion/Cardiogenic tamponade** | | | |
| \| Ahuja et al. 2021 \| \| --- \| | 1.35(1.15-1.58) | <0.001 | 0 |
| \| Brockmeyer et al. 2020 \| \| --- \| | 1.31(1.13-1.51) | <0.001 | 0 |
| \| Faroux et al. 2021 \| \| --- \| | 1.31(1.13-1.51) | <0.001 | 0 |
| \| Fastner et al. 2021 \| \| --- \| | 1.31(1.13-1.52) | <0.001 | 0 |
| \| Kefer et al. 2016 \| \| --- \| | 1.31(1.13-1.52) | <0.001 | 0 |
| \| \| Munir et al. 2021 \| \| --- \| \| \| --- \| --- \| | 1.04(0.77-1.41) | 0.78 | 0 |
| \| Xue et al. 2018 \| \| --- \| | 1.31(1.13-1.52) | <0.001 | 0 |
| **Vascular access complications** | | | |
| \| Ahuja et al. 2021 \| \| --- \| | 1.03(0.81-1.32) | 0.79 | 0 |
| \| Brockmeyer et al. 2020 \| \| --- \| | 1.12(0.91-1.39) | 0.28 | 0 |
| \| Faroux et al. 2021 \| \| --- \| | 1.13(0.90-1.42) | 0.28 | 0 |
| \| Fastner et al. 2021 \| \| --- \| | 1.12(0.90-1.39) | 0.30 | 0 |
| \| Munir et al. 2021 \| \| --- \| | 1.33(0.95-1.87) | 0.10 | 0 |
| **Stroke during in the follow up time** | | | |
| \| Benini et al. 2022 \| \| --- \| | 1.13(0.43-2.95) | 0.81 | 44 |
| \| Brockmeyer et al. 2020 \| \| --- \| | 1.38(0.48-3.92) | 0.55 | 53 |
| \| Faroux et al. 2021 \| \| --- \| | 1.92(0.60-6.22) | 0.27 | 42 |
| \| Fastner et al. 2021 \| \| --- \| | 1.05(0.44-2.50) | 0.92 | 37 |
| \| Luani et al. 2019 \| \| --- \| | 1.03(0.44-2.41) | 0.95 | 35 |
| \| Michlicka-Kłyś et al. 2022 \| \| --- \| | 1.39(0.47-4.07) | 0.55 | 52 |
| \| Xue et al. 2018 \| \| --- \| | 1.91(0.66-5.53) | 0.23 | 42 |
| **Bleeding during in the follow-up time** | | | |
| \| Ahuja et al. 2021 \| \| --- \| | 1.80(1.31-2.49) | <0.001 | 0 |
| \| Benini et al. 2022 \| \| --- \| | 1.65(1.43-1.91) | <0.001 | 0 |
| \| Brockmeyer et al. 2020 \| \| --- \| | 1.67(1.45-1.93) | <0.001 | 0 |
| \| Faroux et al. 2021 \| \| --- \| | 1.64(1.41-1.91) | <0.001 | 0 |
| \| Fastner et al. 2021 \| \| --- \| | 1.66(1.44-1.91) | <0.001 | 0 |
| \| Luani et al. 2019 \| \| --- \| | 1.66(1.44-1.91) | <0.001 | 0 |
| \| Michlicka-Kłyś et al. 2022 \| \| --- \| | 1.67(1.43-1.96) | <0.001 | 2 |
| \| Xue et al. 2018 \| \| --- \| | 1.68(1.46-1.94) | <0.001 | 0 |
| **Mortality during in the following up time** | | | |
| \| Benini et al. 2022 \| \| --- \| | 3.64(1.94-6.82) | <0.001 | 75 |
| \| Brockmeyer et al. 2020 \| \| --- \| | 3.60(1.98-6.54) | <0.001 | 74 |
| \| Faroux et al. 2021 \| \| --- \| | 3.77(1.67-8.47) | 0.001 | 76 |
| \| Fastner et al. 2021 \| \| --- \| | 2.76(2.19-3.49) | <0.001 | 0 |
| \| Luani et al. 2019 \| \| --- \| | 3.36(1.88-6.00) | <0.001 | 74 |
| \| Michlicka-Kłyś et al. 2022 \| \| --- \| | 3.99(2.06-7.72) | <0.001 | 71 |
| \| Xue et al. 2018 \| \| --- \| | 3.90(2.05-7.41) | <0.001 | 73 |
| **Stroke in the end-stage renal disease group** | | | |
| \| Ahuja et al. 2021 \| \| --- \| | 1.03(0.12-8.97) | 0.98 | 52 |
| \| Fastner et al. 2021 \| \| --- \| | 0.96(0.44-2.09) | 0.92 | 6 |
| \| Fink et al. 2023 \| \| --- \| | 1.34(0.40-4.52) | 0.64 | 30 |
| \| Kefer et al.2016 \| \| --- \| | 0.90(0.21-3.88) | 0.89 | 43 |
| \| Munir et al. 2021 \| \| --- \| | 1.35(0.44-4.20) | 0.60 | 23 |
| **In-hospital mortality in the end-stage renal disease group** | | | |
| \| Ahuja et al. 2021 \| \| --- \| | 8.29(4.80-14.30) | <0.001 | 0 |
| \| Fastner et al. 2021 \| \| --- \| | 8.66(5.96-12.59) | <0.001 | 0 |
| \|  \| \| --- \| \| Kefer et al.2016 \| \| | 8.76(6.02-12.74) | <0.001 | 0 |
| \| Munir et al. 2021 \| \| --- \| | 8.57(5.27-13.95) | <0.001 | 0 |
| \| Ueno et al. 2022 \| \| --- \| | 8.57(5.90-12.46) | <0.001 | 0 |
| **Major bleeding events in the end-stage renal disease group** | | | |
| \| Ahuja et al. 2021 \| \| --- \| | 1.60(1.27-2.03) | <0.001 | 0 |
| \| Fastner et al. 2021 \| \| --- \| | 1.61(1.31-1.98) | <0.001 | 0 |
| \| Fink et al. 2023 \| \| --- \| | 1.63(1.33-2.00) | <0.001 | 0 |
| \| Kefer et al.2016 \| \| --- \| | 1.63(1.33-2.01) | <0.001 | 0 |
| \| Munir et al. 2021 \| \| --- \| | 1.84(1.24-2.74) | 0.002 | 0 |
| **Pericardial effusion/tamponade in the end-stage renal disease group** | | | |
| \| Ahuja et al. 2021 \| \| --- \| | 1.49（1.10-2.00） | 0.009 | 0 |
| \| Fastner et al. 2021 \| \| --- \| | 1.52（1.16-2.01） | 0.003 | 0 |
| \| Fink et al. 2023 \| \| --- \| | 1.53（1.17-2.02） | 0.002 | 0 |
| \| Kefer et al.2016 \| \| --- \| | 1.54（1.17-2.03） | 0.002 | 0 |
| \| Munir et al. 2021 \| \| --- \| | 1.97（1.07-3.65） | 0.03 | 0 |
